#  Design and Synthesis of Pyrrolo[2,1-a]Isoquinoline-Based Derivatives as New Cytotoxic Agents

**Published:** 2016

**Authors:** Samaneh Kakhki, Soraya Shahosseini, Afshin Zarghi

**Affiliations:** a *Department of Pharmaceutical Chemistry, School of Pharmacy, Shahid Beheshti Universityof Medical Sciences, Tehran, Iran. *; b *Torbat Heydarieh University of Medical Sciences, Torbat Heydarieh, Iran.*

**Keywords:** Synthesis, 1, 2-diaryl-5, 6-dihydropyrrolo[2, 1-a]isoquinoline, Cytotoxic activity, MTT, Docking study

## Abstract

A new series of anti-cancer agents based on 1,2-diaryl-5,6-dihydropyrrolo[2,1-a]isoquinoline scaffold containing N,N-diethylamino­ethoxy, piperidinyl­ethoxy or morpholinyl­ethoxy group at the *para* position of the C-2 phenyl ring were synthesized and their cytotoxic activities were assessed against several human cancer cell lines including MCF-7 (ER positive breast cancer cell), MDA-MB231 (ER-negative breast cancer cell), T47D (Human ductal breast epithelial tumor cell line), A549 (adenocarcinomic human alveolar basal epithelial cells), and Hela (human cervix adenocarcinoma cells) using MTT assay. Based on results, compounds, 1-(4-(2-(piperidin-1-yl)ethoxy)phenyl)-5,6-dihydro-8,9-dimethoxy-2-phenylpyrrolo[2,1-a]isoquinoline (6a) and 2-(4-(5,6-dihydro-8,9-dimethoxy-2-phenylpyrrolo[2,1-a] isoquinolin-1-yl)phenoxy)-N,N-diethylethanamine (6c) were the most potent cytotoxic compounds and more toxic than the reference compound against T47D cell line, while all the compounds had satisfactory activity against HeLa cell line with mean IC_50_ values ranging from 1.93 to 33.84 µM.

## Introduction

Isoquinoline alkaloids have been reported as an interesting scaffold with considerable pharmacological activities. Recently, [1 ,2 ,4] triazolo[3, 4-a]isoquinolines and pyrrolo[2,1-a]isoquinolines have received much attention as they possess antidepressant, anti-inflammatory, cardiotonic, and potential activity against cancers. They can be also used as positron emission tomography (PET) radiotracers for imaging serotonin uptake sites (1-4). On the other hand, pyrrolo[2, 1-a]isoquinoline serves as intermediates for the synthesis of some antitumor alkaloids such as lamellarins (1) and crysrine (2). These types of alkaloids have been reported to possess antileukemic, tubulin polymerization inhibitory, and antitumor activities. Moreover, related synthetic acetoxy-substituted 5, 6-dihydropyrrolo [2, 1-a]isoquinolines (3) exhibit strong binding affinities for the estrogen (ER) receptors of MCF-7 mammary tumor cell lines (5, 6). In addition, Lamellarin D (1) with isoquinoline scaffold has been reported as a potent Topoisomeras I inhibitor and has the ability to reverse multidrug resistance and induce apoptosis through mitochondria mediated pathway towards a large panel of cancer cell lines (7-10).

Recently, we reported several investigations describing the design, synthesis and molecular modeling studies for a group of 2-phenyl-5, 6-dihydropyrrolo [2,1-a] isoquinolines (4) lacking the basic side chain as anti-cancer agents (11).

**Figure 1:**
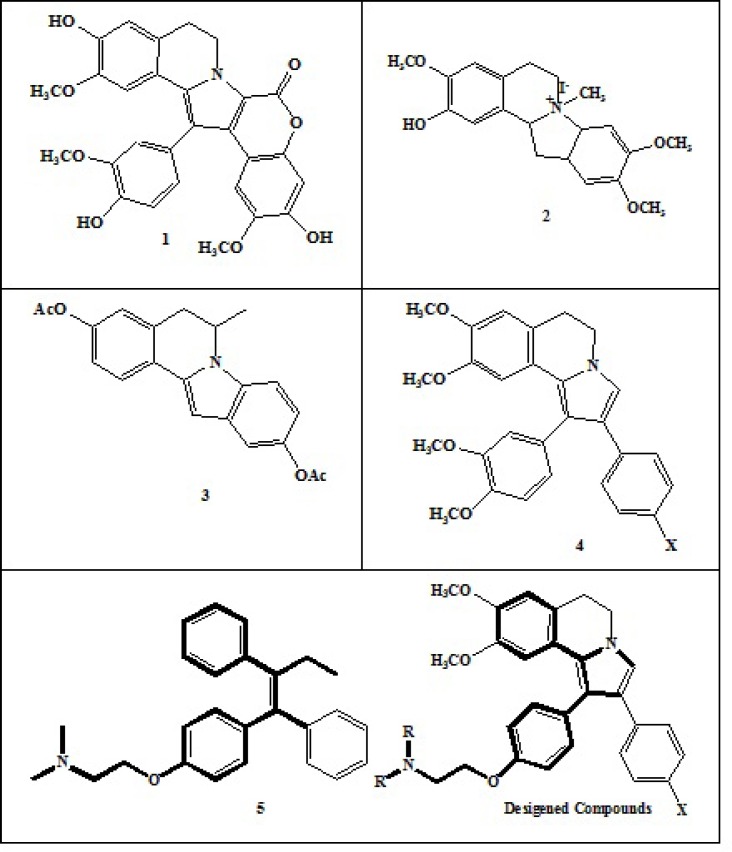
Some representative examples of Pyrrolo[2,1-a]isoquinolineand our designed compounds

Tamoxifen (TAM, 5) is well-known of a family of agents called selective estrogen receptor modulators (SERMs) which is widely used in the clinical management of primary and advanced breast cancer and as a preventive agent against recurrence after surgery since 30 years ago (12, 13). The chemotherapeutic effect of tamoxifen is through prevention of binding of endogenous estrogen to the estrogen receptors within breast cancer cells and slowing the estrogen induced growth. Tamoxifen and many other SERMs could also induce cell death through apoptosis process. Regarding the anticancer properties of pyrrolo[2,1-a]isoquinoline compounds (Figure 1.) and anti breast cancer effects of tamoxifen, the aim of this study was to design new 1, 2-diaryl-5,6-dihydropyrrolo[2,1-a]isoquinoline derivatives possessing pharmacophoric basic side chain that might have expected anticancer activity. The cytotoxic activities of synthesized compounds were evaluated against several human cancer cell lines including MCF-7, MDA-MB231, T47D, A549 and Hela. For designing the new isoquinolines, the vicinal diaryl moiety and the basic side chain (N, N-dialkylaminoethoxyphenyl) was derived from the structure of tamoxifen while the total pyrrolo [2,1-a] isoquinolines resembles the other cytotoxic isoquinoline compounds shown in Figure 1.

## Experimental

*General*

All chemicals and solvents used in this study were purchased from Merck AG and Aldrich except for tamoxifen obtained from (David Bull Labs, UK). Melting points were determined with a Thomas–Hoover capillary apparatus. Infrared spectra were acquired using a Perkin Elmer Model 1420 spectrometer. A Bruker FT-500 MHz instrument (Bruker Biosciences, USA) was used to acquire ^1^H and ^13^C NMR spectra with TMS as internal standard. Chloroform-D and DMSO-D_6_ were used as solvents. Coupling constant (J) values are estimated in hertz (Hz) and spin multiples are given as s (singlet), d (double), t (triplet), q (quartet), m (multiplet), and br (broad). Low-resolution mass spectra were acquired with a MAT CH5/DF (Finnigan) mass spectrometer that was coupled online to a Data General DS 50 data system. The mass spectral measurements were performed on a 6410 Agilent LCMS triple quadruple mass spectrometer (LCMS) with an electrospray ionization (ESI) interface. Microanalyses, determined for C and H, were within ± 0.4% of theoretical values.

*Chemistry*

*General procedure for the preparation of compounds 6a-d*

**Scheme 1:**
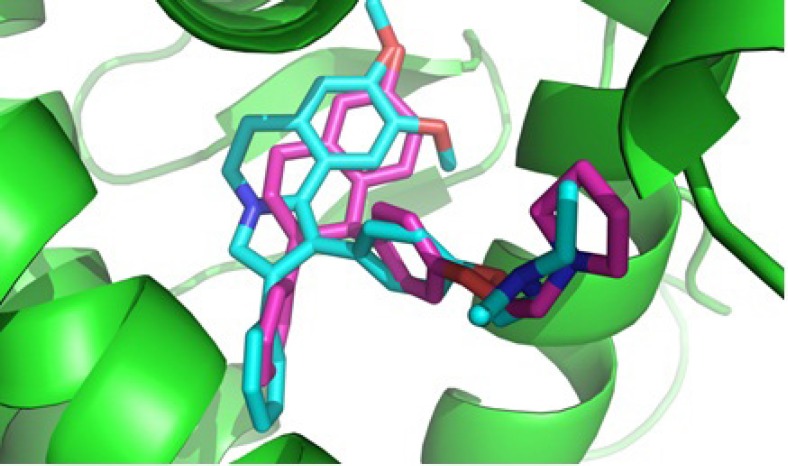
Reagents and conditions

The DHIQ (Dihydroisoquinoline) analogs (compounds 6a-d) were synthesized as depicted in scheme1.

The DHIQ derivatives were prepared starting by 2-(4-hydroxyphenyl)acetic acid which protected with acetic acid using concentrated sulfuric acid as catalyst to give ester 2 (yield: 48%) (14). Amidation of 2 by the treatment of acid chlorides with slight excess of 2-(3, 4-dimethoxyphenyl) ethyl amine afforded desired amide 3 (yield: 65%) (15). According to Bischler-Napieralski reaction, cyclization of amide afforded the 4-((3, 4-dihydro-6, 7-dimethoxyisoquinolin-1-yl)methyl)phenol 4 (yield: 40%) (16). The reaction should be performed by fresh POCl_3_ and using the super dry and high boiling point solvents like toluene and xylene to obtain optimal yields for this series transformation. We also found that the presence of donating groups on β-arylethylamine is an essential need to accelerate the Bischler-Napieralski cyclization. The subsequent reaction of these dihydroisoquinolines with various phenacyl bromide derivatives in anhydrous acetonitrile gave the residue, which was subjected to a plate chromatography to obtain the purified compounds 5a, 5b (yield: 28%-30%). Reaction of 5 with appropriate reagents (N-(2-chloroethyl)-N, N-diethylammonium chloride, (N-(2-chloro ethyl)-piperidinium chloride) and (N-(2-chloro ethyl)-morpholinium chloride) in order to add the basic side chain gave the final products 6a-d (yield: 15-38%) (17). All compounds were pure and stable. The compounds were characterized by ^1^H and ^13^CNMR nuclear magnetic resonance, infrared, mass spectrometry, and CHN analysis.

*2-(4-acetoxyphenyl) acetic acid (2)*

The mixture of (4-hydroxyphenyl) acetic acid dissolved in acetic anhydride using sulfuric acid as catalyst was refluxed for 15 min at 100 °C. After cooling, the mixture was set aside, poured into 200 g crushed ice and the resultant compound (the acetyl conjugate of 1) was obtained as a precipitate, which was filtered, washed with water and drained well. The crude acetyl conjugate (2) was then recrystallized from alcohol to give white crystalline powder. Yield: 48%; White solid; mp: 100 °C; IR (KBr): ν (cm^-1^) 3551-2296 (OH), 1743 (COCH_3_), 1704 (C=O); LC-MS (ESI) *m/z* = 195 (M+1, 100%).

*4-((3, 4-dimethoxyphenethylcarbamoyl) methyl) phenyl acetate (3)*

A mixture of SOCl_2_ (0.43 mL, 6 mmol) and compound 2 (0.58 g, 3 mmol) was stirred at 110 °C for 2 h and then solvent evaporated to give the unstable chloride, which was used in the next reaction without purification. The fresh chloride dissolved in 20 mL dry CH_2_Cl_2_ was added drop wise to a solution containing a mixture of 2-(3,4-dimethoxyphenyl)ethanamine (0.54 mL, 3 mmol) and triethylamine (5.0 mL, 35.8 mmol) in 30 mL dry CH_2_Cl_2_ at ice bath. The resultant mixture was stirred at room temperature for 3 h. After evaporation of CH_2_Cl_2_, the residue was extracted with ethyl acetate (2×30 mL), dried with sodium sulfate and then evaporated; the crude product was crystallized from MeOH to afford colorless crystals (3). Yield: 65%; White solid; mp: 128 ^0^C; IR (KBr): ν (cm^-1^) NH (3307), 1744 (COCH_3_), 1638 (C=O); LC-MS (ESI) *m/z* = 358 (M+1, 100%).

*4-((3, 4-dihydro-6, 7-dimethoxyisoquinolin-1-yl) methyl) phenol (4)*

According to Bischler–Napieralski reaction (9, 16), to a mixture of above intermediate 3 (1.03 g, 3 mmol) in dry toluene, fresh phosphorous oxychloride (2.8 mL, 30 mmol) was added and the reaction mixture was refluxed at 110 °C for 4 h. To the cold mixture was added cold petroleum ether (20 mL). The suspension was cooled into ice bath for another 2 h, the supernatant liquid was decanted and the remaining crude product was dissolved in acetone/water (10/20 mL) solution, neutralized with strong aqua ammonia to pH 9–10 to produce precipitate. The crude products were recrystallized from methanol several times to afford final products (4). Yield: 40%; Yellow solid; mp: 186 °C; IR (KBr): ν (cm^-1^)3397-2444 (OH), 1643 (C=N); LC-MS (ESI) *m/z* = 298 (M+1, 100).

*4-(5, 6-dihydro-8, 9-dimethoxy-2-phenylpyrrolo [2, 1-a] isoquinolin-1-yl) phenol (5a)*

The phenacyl bromide (5 g, 25 mmol) and NaHCO_3_ (8 g, 100 mmol) was added to a solution of dihydroisoquinoline hydrochloride 4 (7 g, 25 mmol) in anhydrous acetonitrile (15 mL) at room temperature. The mixture was stirred under refluxing for 12 h. The solvent was removed in vacuum. The residue was dissolved in CHCl_3_ (40 mL), washed with brine (20 mL) and dried over Na_2_SO_4_. The solvent was evaporated to obtain a residue, which was purified by plate chromatography to give pure green crystalline powders (5a).Yield: 28%; Green solid; mp: 210 °C; IR (KBr): ν(cm^-1^) 1151, 1233, 1282, 1509, 1562; LC-MS (ESI) *m/z* = 398 (M+1, 100%); ^13^CNMR (300 MHz/CDCl_3_): 29.3, 44.4, 55.2, 107.8, 111.0, 113.4, 115.3, 117.9, 118.1, 122.3, 123.5, 124.1, 126.2,128.1, 128.4, 128.9, 129.2, 132.3, 146.8, 147.5, 155.1; Anal. Calcd. for C_26_H_23_NO_3_: C, 78.57; H, 5.83; N, 3.52. Found: C, 78.77; H, 5.96; N, 3.77.

*4-(5, 6-dihydro-8, 9-dimethoxy-2--(4-methoxyphenyl) pyrrolo [2, 1-a] isoquinolin-1-yl) phenol (5b)*

The 2-bromo-1-(4-methoxyphenyl) ethanone (5.6 g, 25 mmol) and NaHCO_3_ (8 g, 100 mmol) was added to a solution of dihydroisoquinoline hydrochloride 4 (7 g, 25 mmol) in anhydrous acetonitrile (15 m) at room temperature. The mixture was stirred under refluxing for 12 h. The solvent was removed in vacuum. The residue was dissolved in CHCl_3_ (40 mL), washed with brine (20 mL) and dried over Na_2_SO_4_. The solvent was evaporated to obtain a residue, which was purified by plate chromatography to afford pure green crystalline powder (5b).Yield: 30%; Green solid; mp: 218 °C; IR (KBr): ν(cm^-1^) 1119, 1243, 1263, 1490, 1512; LC-MS (ESI) *m/z* = 428 (M+1, 100); ^13^CNMR (300 MHz/CDCl_3_): 29.4, 44.6, 55.1, 55.8, 107.4, 111.1, 113.5, 115.4, 117.7, 118.2, 122.4, 123.8, 123.9, 126.2, 128.2, 128.8, 129.0, 132.4, 146.5, 147.2, 154.5, 157.3; Anal. Calcd. for C_27_H_25_NO_4_: C, 75.86; H, 5.89; N, 3.28. Found: C, 75.67; H, 5.56; N, 3.40.

*General procedure for synthesis of 8, 9-dimethoxy-2-phenyl1-(N, N-dialkylaminoethoxy) phenyl)-5, 6-dihydropyrrolo [2, 1-a]isoquinolin (6a-d)*

(4.42) mmol of 5a and 5b were dissolved in 40 mL acetonitrile and 5.8 g K_2_CO_3_ was added, while stirring. 4 equivalents of N-(2-chloroethyl)piperidinium chloride, N-(2-chloroethyl) morpholinium chloride and N-(2-chloroethyl)-N, N-diethylammonium chloride were added subsequently and the reaction mixture was refluxed for 24 h. The inorganic material was filtered off and filtrate was evaporated under vacuum, dissolved in DCM and washed with brine. The organic phase was dried with sodium sulfate and evaporated. The oily residue was purified using column chromatography with a mobile phase of CHCl_3_, to obtain a pure yellow crystalline powder.

*1-(4-(2-(piperidin-1-yl)ethoxy)phenyl)-5, 6-dihydro-8, 9-dimethoxy-2-phenylpyrrolo[2, 1-a] isoquinoline (6a)*

Yield: 38%; Yellow solid; mp: 100 °C; IR (KBr): ν (cm^-1^) 1119, 1243, 1263, 1490, 1512; LC-MS (ESI) *m/z* = 509 (M+1, 100); ^1^HNMR (500MHz/CDCl_3_): δ ppm 1.38 (m, 2 H, piperidine CH_2_), 1.49 (m, 4H, piperidine CH_2_), 2.44 (m, 4H, piperidine CH_2_), 2.63 (s, 2H, -CH_2_CH_2_N-), 2.96 (t, 2H, -CH_2_N-, *J = *6.43H_Z_), 3.21 (s, 3H, 8-OCH_3_), 3.71 (s, 3H, 9-OCH_3_), 4.07 (t, 4H, -CH_2_O- & -CH_2_CH_2_N-), 6.35 (s, 1H, pyrrole), 6.86 (s, 1H, 7-Ar-H), 6.96 (d, 2H, (4-(2-piperidin-1-yl)ethoxy)phenyl), H_3_&H_5_, *J = *8.8H_Z_), 7.05-7.16 (m, 6H, (4-(2-piperidin-1-yl)ethoxy)phenyl), H_2_&H_6_)& phenyl & 10-Ar-H); ^13^CNMR (300 MHz/CDCl_3_): 23.9, 25.7, 29.3, 44.5, 54.9, 55.7, 57.7, 65.7, 107.3, 110.9, 114.5, 118.0, 118.3, 122.2, 123.8, 124.1, 125.0, 126.2, 127.6, 127.8, 128.9, 132.1, 135.5, 146.5, 147.1, 157.4; Anal. Calcd. for C_33_H_36_N_2_O_3_: C, 77.92; H, 7.13; N, 5.51. Found: C, 78.10; H, 7.26; N, 5.40.

*1-(4-(2-morpholinoethoxy) phenyl)-5, 6-dihydro-8, 9-dimethoxy-2-phenylpyrrolo [2, 1-a] isoquinoline(6b)*

Yield: 24%; Yellow solid; mp: 90 °C; IR (KBr): ν (cm^-1^) 1121, 1178, 1241, 1269, 1500; LC-MS (ESI) *m/z* = 511(M+1, 100); ^1^HNMR (500MHz/CDCl_3_): δppm 1.68 (s, 2H, -CH_2_CH_2_N), 2.7-2.9 (m, 4H, morpholine CH_2_), 3.08 (t, 2H, -CH_2_N), 3.40 (s, 3H, 8-OCH_3_), 3.88 (m, 4H, morpholine CH_2_), 3.89 (s, 3H, 9-OCH_3_), 4.13 (t, 4H, -OCH_2 _& -CH_2_CH_2_N), 6.61 (s, 1H, pyrrole), 6.72 (s, 1H, 7-Ar-H), 6.88 (s, 1H, 10-Ar-H), 6.92 (d, 2H, (4-(2-morpholine-1-yl)ethoxy)phenyl), H_3_&H_5_, *J = 8.4 *H_Z_), 7.19 -7.21 (m, 5H, phenyl), 7.28 (d, 2H, 4-(2-morpholine-1-yl)ethoxy)phenyl), H_2_&H_6_*, J = *9.2H_Z_); ^13^CNMR (300 MHz/CDCl_3_): 29.3, 44.5, 54.0, 55.0, 55.7, 57.5, 65.6, 66.8, 107.2, 111.0, 113.3, 114.5, 117.6, 118.1, 122.3, 123.8, 126.1, 128.0, 128.7, 129.2, 132.1, 146.5, 147.1, 157.3; Anal. Calcd. for C_32_H_34_N_2_O_4_: C, 57.27; H, 6.71; N, 5.49. Found: C, 56.98; H, 6.96; N, 5.60.

*2-(4-(5, 6-dihydro-8, 9-dimethoxy-2-phenylpyrrolo [2, 1-a] isoquinolin-1-yl)phenoxy)-N, N-diethylethanamine (6c)*

Yield: 15%; Yellow solid; mp: 99 °C; IR (KBr): ν (cm^-1^) 1043, 1134, 1253, 1272, 1499, 1521; LC-MS (ESI) *m/z* = 497(M+1, 100); ^1^HNMR (500MHz/CDCl_3_): δppm 1.2 (m, 6H, CH_3_), 2.73 (m, 4H, -CH_2_N), 2.96 (m, 2H, -CH_2_N), 3.05 (t, 2H, -CH_2_CH_2_N, *J = *6H_Z_), 3.38 (s, 3H, 8-OCH_3_), 3.87 (s, 3H, 9-OCH_3_), 4.10 (t, 4H, -OCH_2_& -CH_2_CH_2_N), 6.60 (s, 1H, pyrrole), 6.70 (s, 1H, 7-Ar-H), 6.86 (s, 1H, 10-Ar-H), 6.90 (d, 2H, (N, N-diethylethenamine)phenoxy), H_3_&H_5_, *J = *8.5H_Z_), 7.10 -7.20 (m, 5H, phenyl), 7.28 (d, 2H, (N, N-diethylethenamine)phenoxy), H_2_&H_6_, *J = *8.5H_Z_); ^13^CNMR (300 MHz/CDCl_3_): 11.8, 29.4, 44.6, 47.8, 51.7, 55.1, 55.8, 66.5, 107.4, 111.0, 114.6, 118.4, 122.4, 123.0, 124.2, 125.1, 126.3, 127.7, 127.9, 128.9, 132.2, 135.636, 146.6, 147.3, 157.6; Anal.Calcd. for C_32_H_36_N_2_O_3_: C, 77.39; H, 7.31; N, 5.64. Found: C, 77.59; H, 7.08; N, 5.41.

*1-(4-(2-morpholinoethoxy) phenyl)-5, 6-dihydro-8, 9-dimethoxy-2-(4-methoxyphenyl)pyrrolo[2, 1-a] isoquinoline(6d)*

Yield: 23%; Yellow solid; mp: 90 °C; IR (KBr): ν (cm^-1^) 1130, 1255, 1275, 1502, 1523; LC-MS (ESI) *m/z* = 541 (M+1, 100); ^1^HNMR (400MHz/CDCl_3_): δ ppm 2.67 (t, 2H, morpholine –CH_2_N), 2.94 (t, 2H, -CH_2_N), 3.20 (s, 3H, (4-methoxy-phenyl)), 3.56 (t, 4H, morpholine -OCH_2_), 3.66 (s, 3H, 8-OCH_3_), 3.70 (s, 3H, 9-OCH_3_), 4.02 (t. 4H, -OCH_2_), 4.07 (t, 2H, -CH_2_CH_2_N), 6.34 (s, 1H, pyrrole), 6.71 (d, 2H, (4-(2-morpholine-1-yl)ethoxy)phenyl), H_3_&H_5_, *J = *8.8H_Z_), 6.84 (s, 1H, 7-Ar-H), 6.95 (d, 2H, 4-(2-morpholine-1-yl)ethoxy)phenyl), H_2_&H_6_, *J = *8.8H_Z_), 7.00 (s, 1H, 10-Ar-H), 7.01 (d, 2H, 4-(OCH_3_-phenyl, H_3_&H_5_, *J = *8.8 H_Z_), 7.12 (d, 2H, 4-(OCH_3_-phenyl, H_2_&H_6_, *J = *8.8 H_Z_);^13^CNMR (300 MHz/CDCl_3_): 29.4, 44.6, 54.1, 55.1, 55.8, 57.6, 65.8, 66.9, 107.4, 111.1, 114.6, 118.2, 118.3, 122.3, 123.9, 124.2, 125.2, 126.4, 127.8, 128.0, 129.2, 132.2, 135.6, 146.7, 147.3, 157.4; Anal. Calcd. for C_33_H_36_N_2_O_5_: C, 73.31; H, 6.71; N, 5.18. Found: C, 73.52; H, 6.99; N, 4.96.

*Cytotoxicity*

To determine the cytotoxicity of the pyrrolo[2,1-a]isoquinolines-based derivatives, five human tumor cell lines were used; MCF-7 (breast cancer cell), MDA-MB231(without estrogen receptors), T47D (Human ductal breast epithelial tumor cell line), A549 (adenocarcinomic human alveolar basal epithelial cells), and Hela (human cervix adenocarcinoma cells) cell lines were purchased from Iranian Biological Resource Center (IBRC), Tehran, Iran (18-20). 

The cells were cultured in RPMI1640 medium at 37 °C under 5% CO_2_ supplemented with 10% fetal bovine serum (FBS), 100 U/mL penicillin and 100 µg/mL streptomycin. Cell viability was assayed by using a MTT method which is based on the reduction of 3-(4, 5-dimethylthiazol-2-yl)-2, 5-diphenyltetrazolium bromide (MTT) dye to purple formazan crystals by mitochondrial succinate dehydrogenase enzyme in living cells. The cells were seeded into 96-well plates at a concentration of 10^4^ cells/well and allowed to incubate for 24 h. 

**Table 1:** In vitro antiproliferative activity of compounds **6a-d** based on MTT assay

**Compounds**	**R**	**X**	**MCF-7** **IC**_50_ ** (μM)** a	**T47D** **IC**_50_ ** (μM)**	**MDA-MB-231** **IC**_50_ ** (μM)**	**HeLa** **IC**_50_ ** (μM)**	**A549** **IC**_50_ ** (μM)**
6a	Piperidine	H	12.30	5.18	15.89	13.63	22.20
6b	Morpholine	H	>100	>100	>100	1.93	57.64
6c	Diethylamine	H	4.73	2.34	19.87	6.93	20.14
6d	Morpholine	OMe	>100	>100	>100	33.84	>100
Tamoxifen			12.67	17.89	13.26	14.91	19.69

a : IC_50_: drug concentration that inhibits cell growth by 50%.

**Figure 2:**
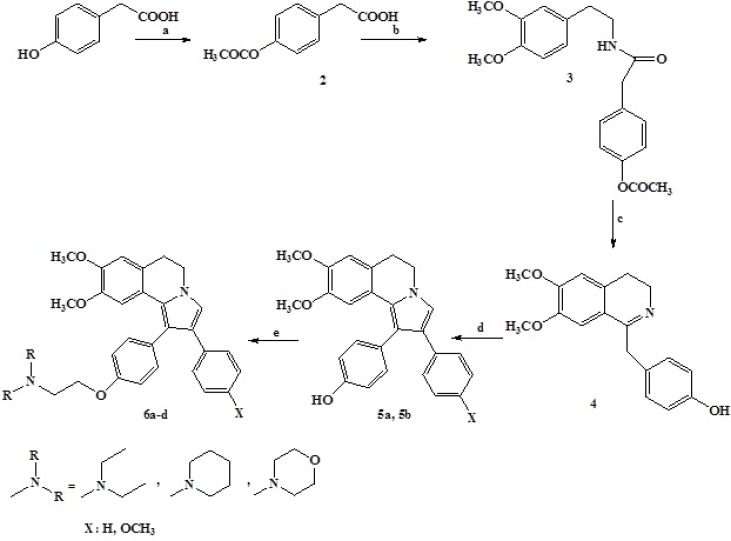
Docking **6c** in the active site of human ERα. Hydrogen atoms have been removed to improve clarity

**Figure 3:**
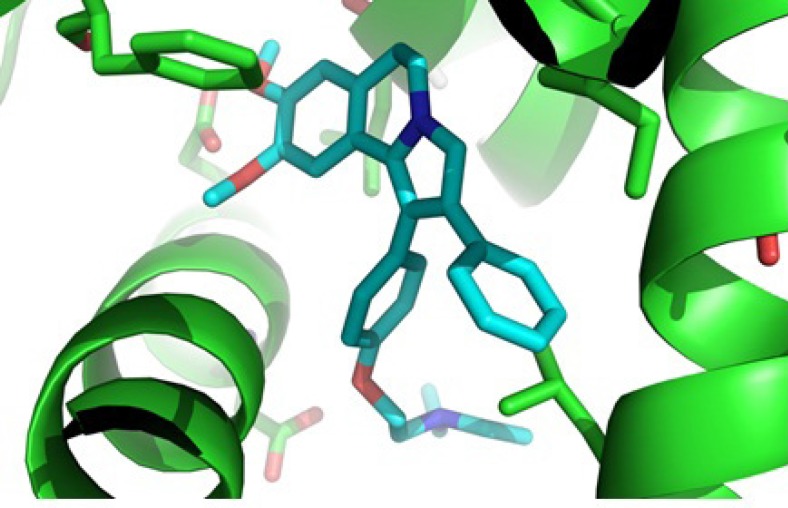
Good superimposition of the dihydroisoquinoline moiety of the synthesized compound **6c** with the lasofoxifen

The cells were incubated with increasing concentrations of test compounds for 48 h. At the end of each treatment period, 10 μL of MTT (5 mg/mL in PBS) was added to each well and the microplate was incubated at 37 °C for 4 h. The medium with MTT was removed and 100 μL DMSO was added to each well to dissolve the insoluble formazan crystals. Plates were incubated for 20 min at 37 °C and the optical densities were read at 570 nm with a reference wavelength of 630 nm as background using a spectrophotometer plate reader (Infinite® M200, TECAN) (21). Tamoxifen was also used as positive control and DMSO as the solvent of the test compounds. Data are presented as the mean of triplicate number of living cells and their capacity to reduce samples. IC50 was calculated by calibration curve using Prism software**.**

*Molecular modeling (docking) studies*

Docking studies were performed using AutodockVina software (22). The coordinates of the X-ray crystal structure of the selective estrogen receptor modulator lasofoxifen bound to the human estrogen receptor α was obtained from the RCSB Protein Data Bank (2OUZ) and hydrogens were added, Kollman charge was calculated and non-polar hydrogens were deleted. The ligand molecule was constructed using the Hyperchem 8.0.3 and was energy minimized using AMBER MM force field, Polak-Ribier algorithm for 1000 interactions reaching a convergence of 0.01 Kcal/mol Å. A grid box of 24-24-24 Å with the central X-Y-Z coordinates of X: 30.6130 Y: -1.2140 Z: 27.6360 were built for calculation of the energy map. At the end of docking process, conformations having optimal docking energy were visualized using Viewer Lite 5.0 software, residues with atoms greater than 7.5 Å from the docking box were removed for efficiency and the distances between atoms of ligand and amino acids in the active site were calculated. For docking validation, lasofoxifen was docked in the active site of ERα with exactly similar conditions and the docked conformation having lowest docking energy was aligned with lasofoxifen in crystallography with ERα (2OUZ), using Pymolsoftware and also RMSD acquired was 1.74 Å showing that the docking method was valid (23).

## Results and discussion

To determine the effect of synthesized compounds on the viability of breast cancer cells in vitro, ER-α-positive MCF7 and T-47D cells (containing medium to high levels of estrogen receptors), ER-α-negative MDA-MB-231 (without estrogen receptors) and two different cancer cell lines such as A549 (adenocarcinomic human alveolar basal epithelial cells) and Hela (human cervix adenocarcinoma cells) were used in MTT assay. To indicate the anti-proliferative activities of the designed compounds mediated through hormone-dependent or hormone-independent mechanisms, the cells were treated with increasing concentrations of synthesized compounds (0–100 μM) and TAM (tamoxifen) (0–100 μM) as a reference drug.

The results of MTT assay are shown in Table 1. According to results, the synthesized compound 6b was the most cytotoxic compound against HeLa cell line while it was inactive against all types of breast cancer cell lines. The compounds 6a and 6c were cytotoxic against all cancer cell lines and showed the most potency against T47D cell line which contains high amounts of estrogen receptors in comparison with those of MCF7 and MDA-MB-231. This may be explained due to the ability of synthesized compounds to blockade estrogen receptors in breast cancer cell lines. In addition, our results showed that the presence of hydrophobic side chain such as piperidinyl or ethoxy increased cytotoxicity in comparison with compounds 6b and 6d containing polar morpholine group as polar side chain. However, our results showed that the synthesized compounds had also moderate to good cytotoxic activities on MDA-MB-231 cell line which indicated that other anticancer mechanisms may be involved in addition to blockade estrogen receptors in breast cancer cell lines.

Based on our MTT assay and structure similarity between designed 1, 2-diaryl-5, 6-dihydropyrrolo [2,1-a] isoquinoline compounds 6a-d and tamoxifen, it could be assumed that one of the mechanisms for cytotoxic activity of compounds 6a-d on breast cancer cell lines is mediated through estrogen receptors. Therefore, the orientation of 2-(4-(5, 6-dihydro-8, 9-dimethoxy-2-phenylpyrrolo[2,1-a] isoquinolin-1-yl)phenoxy)-N, N-diethylethanamine 6c as the most potent compound against T47D, in the estrogen receptor α (ERα) active site was examined by a docking experiment (Figure 2). This molecular modeling study showed that compound 6c was well bound into the active site of ERα so that the *N* atom of the tertiary amino group of the basic side chain (diethylaminophenoxy) is in the vicinity of the oxygen of carboxylate group of Asp^351^ (distance = 4.01 Å) and is capable of binding to this amino acid. The methoxy group on phenyl ring is also in the vicinity of Glu^353^ so that the *O *atom of methoxy group is in a 2.6 Å distance from the O*H* of carboxylic group in Glu^353^ (24). In addition, molecular modeling studies (Figure 3.) showed the good superimposition of compound 6c with lasofoxifen as a crystallography compound in the estrogen receptor active site. These data together with biological results are in agreement that one of the mechanisms of cytotoxic activity of compounds 6a and 6c on breast cancer cell lines might be mediated through acting on estrogen receptors.

## Conclusion

This study indicates that all synthesized compounds showed significant cytotoxicity against HeLa cell line while compounds 6a and 6c demonstrated cytotoxicity against all types of breast cancer lines. In addition, modifications on the basic side chain of 1, 2-diaryl-5, 6-dihydropyrrolo [2,1-a] isoquinoline scaffold had a significant influence on the cell cytotoxicity. 
